# Invasive Gastric Candidiasis With Concurrent Clostridioides difficile Colitis: A Case Report and Review of the Literature

**DOI:** 10.7759/cureus.88115

**Published:** 2025-07-16

**Authors:** Sevag Hamamah, Garrett Teskey, Wesley Chow, Evan Wilder, Laya Reddy, Faizi Hai

**Affiliations:** 1 Internal Medicine, Scripps Mercy Hospital, San Diego, USA; 2 Gastroenterology, Scripps Green Hospital/Scripps Clinic, La Jolla, USA; 3 Infectious Disease, Scripps Mercy Hospital, San Diego, USA; 4 Gastroenterology, Scripps Mercy Hospital, San Diego, USA

**Keywords:** clostridioides difficile, gastric ulcers, gastroenterology and endoscopy, infectious disease, invasive gastric candidiasis

## Abstract

Invasive gastric candidiasis is a rare fungal infection of the stomach, often arising in the setting of immunosuppression, critical illness, mucosal barrier disruption, gut microbial alterations, or antibiotic use. Though *Candida spp. *are normal flora within the gastrointestinal tract, compromised host defenses can contribute to overgrowth and invasion of the fungal species into deeper tissues. We report a case of an 89-year-old man presenting with concurrent upper gastrointestinal bleeding from diffuse gastric ulcers secondary to invasive gastric candidiasis and diarrhea resulting from *Clostridioides difficile *colitis. The pathophysiology, diagnosis, and management of invasive candidiasis as well as interactions between gastrointestinal candidiasis and *Clostridioides difficile *infection are discussed. Overall, this case highlights the interplay between antibiotic use, gut barrier translocation, interactions between microorganisms, and worsening infectious disease in an elderly patient. Similarly, it stresses the importance of maintaining a high index of suspicion for fungal infections in patients with large atypical appearing gastric ulcers, even in the absence of overt immunosuppression.

## Introduction

*Candida* is a genus of fungi that is considered normal flora of the skin, mouth, gastrointestinal (GI), and genitourinary tracts [1]. Under normal physiological circumstances, *Candida* *spp. *are harmless commensal organisms that are suppressed by host immune factors, healthy microbial flora, and intact mucosal barriers [1]. When host defense mechanisms are compromised,* Candida spp.* may overgrow and confer infections, ranging from superficial mucosal disease to severe systemic illness [2].

Invasive candidiasis occurs when *Candida spp.* invade mucosal barriers, permeating into deep tissues or into the bloodstream, which are termed deep-seated candidiasis and candidemia, respectively [2]. In immunosuppressed individuals with invasive candidiasis, the following sites of involvement have been characterized via culture and histopathological examination: lung (79%), serum (37%), GI tract (35%), kidney (34%), liver (20%), and spleen (19%) [3]. The exact distribution of involvement within the GI tract varies between studies; however, the esophagus is the most commonly involved site, with the stomach, small intestines, and large intestine affected to lesser extents [4]. Risk factors for mucosal and serum invasion of *Candida spp.* include immunosuppressive therapy, immunodeficiency, neutropenia, major intra-abdominal surgery, prolonged intensive care unit stay, broad-spectrum antibiotic use, and diabetes mellitus [2,5].

Invasive gastric candidiasis is a rare entity that can present with nonspecific symptoms such as abdominal pain, nausea, vomiting, or GI bleeding, which may delay diagnosis [6]. In severe forms, findings may consist of ulceration, thick white plaques, and even perforation [6]. We report a fatal case of an 89-year-old male presenting with concurrent acute upper GI bleeding and diarrhea, in which gastric ulcers secondary to invasive gastric candidiasis were confirmed on biopsy with concurrent *Clostridioides difficile* colitis identified through laboratory testing. This case highlights the interplay between antibiotic use, gut barrier translocation, microorganisms, and worsening infectious disease in an elderly patient.

## Case presentation

An 89-year-old man with a history of type 2 diabetes mellitus, hypertension, and iron deficiency anemia presented with one week of right upper quadrant abdominal pain, nausea, and vomiting. Pertinent exam findings included moderate right upper quadrant tenderness with positive Murphy’s sign. On laboratory evaluation, he was noted to have leukocytosis with a white blood cell (WBC) count of 15.4 K/mcL and anemia with hemoglobin (Hgb) of 11.2 g/dL (Table 1).

**Table 1:** Trend of Complete Blood Count (CBC) with Differential Over Two Hospitalizations. Initial hospitalization denotes patient's initial presentation with right upper quadrant abdominal pain, where the patient was found to have acute cholecystitis. Hospital days indicate patient's second hospitalization four weeks later for invasive gastric candidiasis, *Clostridioides difficile *colitis, and extended-spectrum beta-lactamase *Escherichia coli *bacteremia. WBC, white blood cell; Hgb, hemoglobin; HD, hospital day; K/mcL, thousands per microliter; g/dL, grams per deciliter; %, percent.

Lab Value	Initial Admission	HD1	HD3	HD4	HD5	HD8	Reference Range
WBC (K/mcL)	15.4	22.3	33.3	24.2	17.5	16.0	3.4 – 11.0
Hgb (g/dL)	11.2	8.9	8.8	7.6	7.3	7.5	13.0 – 17.1
Platelets (K/mcL)	246	235	233	251	214	255	150 - 425
Immature Granulocyte %	0.7	1.3	4.2	2.0	1.3	3.0	0.0 – 1.2
Neutrophil %	88.6	88.9	88.1	84.2	81.5	76.9	38.0 – 74.0
Lymphocyte %	4.8	5.3	3.6	5.1	7.8	12.7	16.0 – 48.0
Monocyte %	5.3	4.4	3.7	3.9	4.9	5.7	4.9 – 12.5
Eosinophil %	0.5	0.0	0.3	0.8	1.7	1.5	0.0 – 9.5
Basophil %	0.1	0.1	0.2	0.1	0.1	0.2	0.0 – 0.09

**Figure 1:**
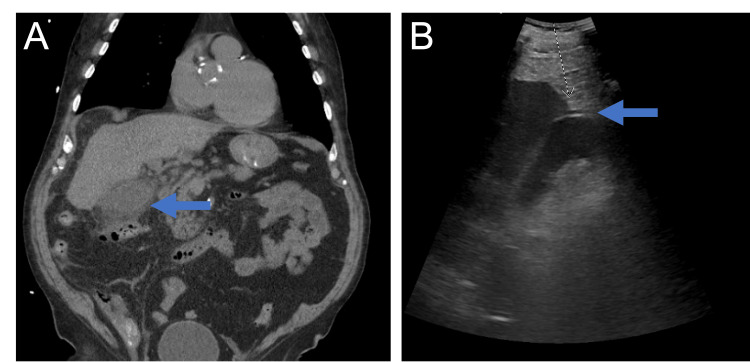
Computed Tomography (CT) of the Abdomen and Right Upper Quadrant (RUQ) Ultrasound Demonstrating Concern for Cholecystitis. Left: CT abdomen and pelvis showing distended gallbladder with wall thickening and pericholecystic inflammation. The arrow points to the inflamed gallbladder. Right: RUQ ultrasound showing evidence of cholelithiasis. The arrow points to a gallstone.

During the initial admission, the patient was found to have a distended gallbladder with diffuse pericholecystic inflammation concerning for acute cholecystitis seen on computed tomography (CT) of the abdomen (Figure 1). Right upper quadrant ultrasound identified evidence of cholelithiasis. The hepatobiliary iminodiacetic acid (HIDA) scan confirmed diagnosis of cholecystitis, and robotic-assisted cholecystectomy was subsequently performed. Intraoperative findings showed a gangrenous gallbladder with mural abscess; however, the gallbladder was not cultured to identify the causative microorganism. During this time, the patient was treated with ceftriaxone and metronidazole, eventually being discharged on a five-day course of amoxicillin-clavulanate for a total eight-day course of antibiotics.

**Figure 2:**
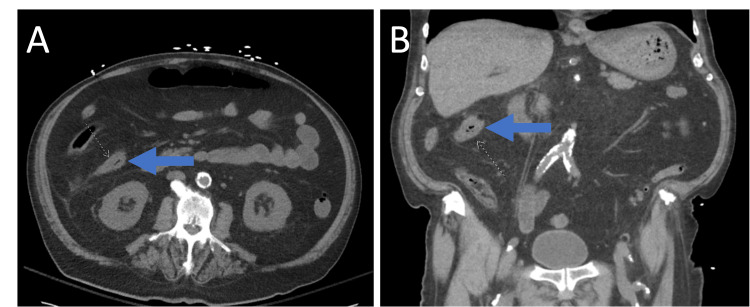
Computed Tomography (CT) of the Abdomen Showing Ascending Colonic Wall Thickening. Arrows pointing to the thickened wall of the ascending colon. Axial (left) and coronal (right) views are shown.

Four weeks later, the patient returned after having two syncopal episodes, coffee-ground emesis, melena, right-sided abdominal pain, and multiple episodes of diarrhea within a one-week period. There was no history of prior non-steroidal anti-inflammatory drug (NSAID) use and the patient was not previously on a proton pump inhibitor (PPI). Vital signs showed mild hypertension but were otherwise stable. On examination, he exhibited conjunctival pallor, dry mucous membranes, and mild diffuse abdominal tenderness. No peritoneal signs such as guarding or rebound tenderness were present. Laboratory findings on hospital day 1 included worsened leukocytosis with WBC 22.3 K/mcL, normocytic anemia with Hgb 8.9 g/dL and MCV 93 fL, elevated blood urea nitrogen (BUN) 108 mg/dL, creatinine 2.10 mg/dL, C-reactive protein (CRP) 140 mg/dL, and erythrocyte sedimentation rate (ESR) 97 mm/hr. Human immunodeficiency virus (HIV) testing was negative. Abdominal CT revealed ascending colonic wall thickening (Figure 2). Blood cultures grew extended-spectrum beta-lactamase (ESBL)-resistant *Escherichia coli*, and stool testing was positive for *Clostridioides difficile*. He was started on empiric treatment with meropenem and oral vancomycin.

**Figure 3:**
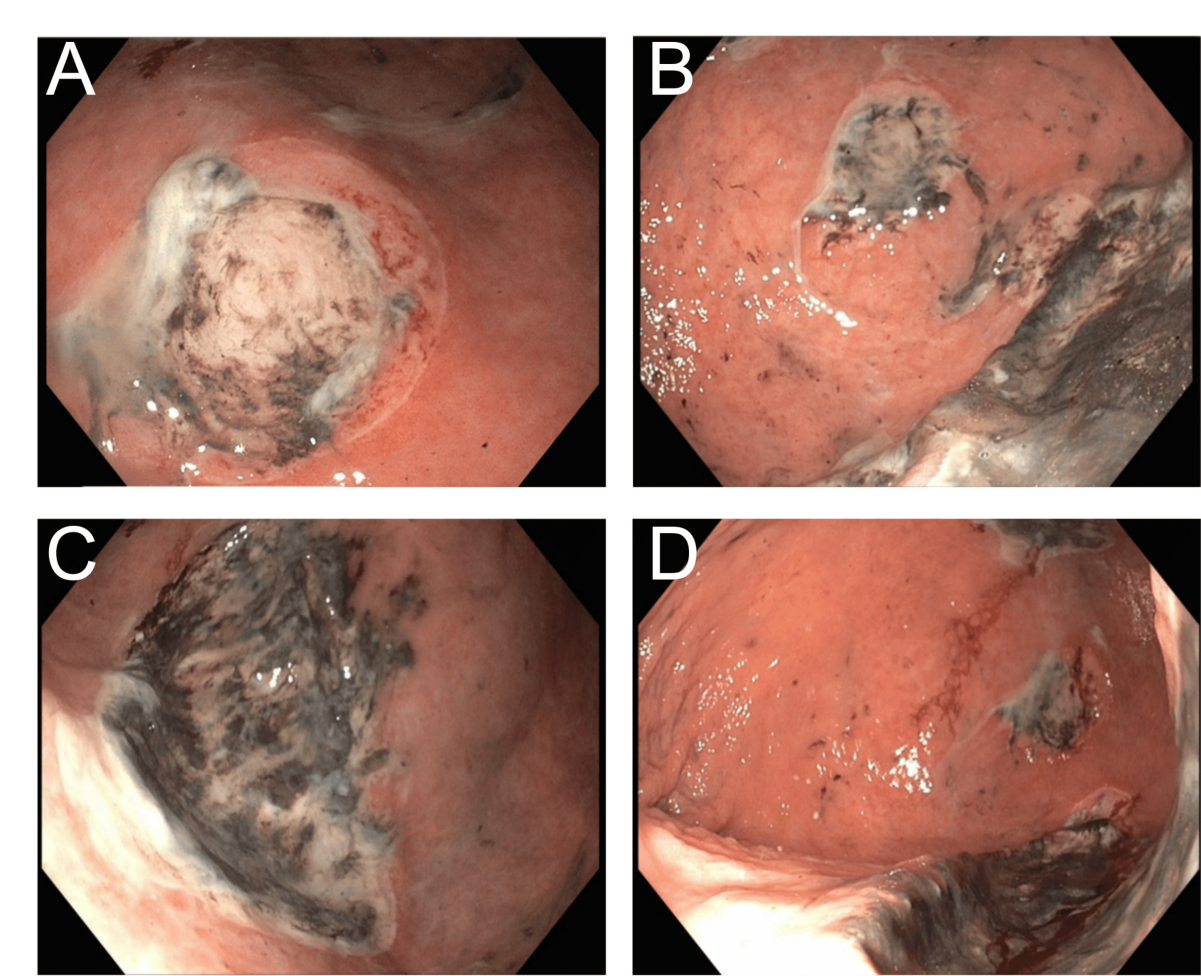
Endoscopic Views of Diffuse Gastric Ulcers. Multiple large (2–10 cm) atypical appearing, deep cratered gastric ulcers with adherent dark material along the lesser and greater curvatures are seen on esophagogastroduodenoscopy.

Gastroenterology was consulted on admission for melena. Esophagogastroduodenoscopy revealed multiple large (2-10 cm) atypical appearing, deep cratered gastric ulcers with adherent dark material along the lesser and greater curvatures with otherwise normal esophagus and duodenum (Figure 3).

**Figure 4:**
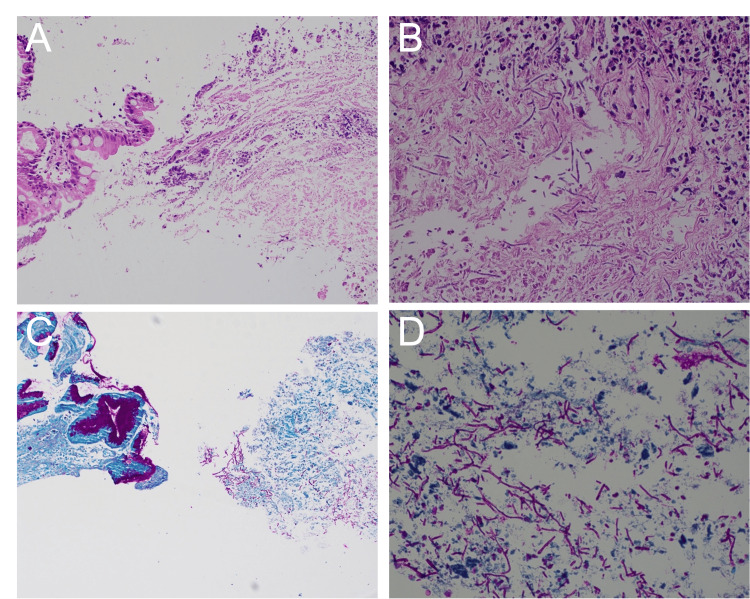
Gastric Biopsies Stained with Hematoxylin and Eosin (H&E) and Periodic Acid-Schiff (PAS). Top Left: H&E (low magnification) shows intestinal metaplasia with focal epithelial damage and inflammation. Top Right: H&E (high magnification) reveals acute-on-chronic inflammation, necrosis, and fungal invasion. Bottom Left: PAS stain highlights pseudohyphae and yeast forms infiltrating mucosa. Bottom Right: PAS (high magnification) showing abundant magenta-stained fungal elements within inflamed tissue.

Biopsies of the gastric ulcers demonstrated marked chronic active gastritis with ulceration, intestinal metaplasia, and necro-inflammatory debris with abundant adjacent fungal pseudohyphae highlighted by Periodic Acid-Schiff (PAS) staining (Figure 4). Gastrin, chromogranin, *Helicobacter pylori*, Cytomegalovirus, and Acid-Fast Bacilli stains were all negative. Given confirmation of *Candida *infection through histopathology and negative stains for other infectious pathology, further PCR testing and culture were not performed.

Infectious disease was consulted, and intravenous fluconazole was initiated for the diffuse gastric ulcers. Despite clinical improvement and plans for discharge, the patient unexpectedly passed away due to cardiac arrest on hospital day 8 in the setting of necrotizing gastritis, ESBL bacteremia, and *Clostridioides difficile *colitis. Given the patient's do-not-resuscitate (DNR) code status, cardiopulmonary resuscitation was not performed. Autopsy was declined.

## Discussion

Pathologic gastric candidiasis is a rare occurrence in individuals with the absence of traditional immunosuppressive risk factors. While *Candida spp.* are part of the normal GI microbiota, mucosal disruption can allow colonizing *Candida* to transition into a pathogenic state. This patient lacked traditional risk factors for fungal infections, such as HIV, malignancy, toxic ingestion, or antacid use [7]. His older age, recent course of antibiotics, recent hospitalization, severity of illness, and diabetes may have been risk factors causing relative immunosuppression, likely predisposing him to invasive gastric candidiasis, ESBL-resistant *Escherichia coli *bacteremia, and *Clostridioides difficile* colitis.

Within the literature, cases of invasive gastric candidiasis remain underreported. Of the reported cases in the last 15 years [6,8-20], *Candida albicans* was the most frequently isolated species; however, other *Candida spp.* such as *tropicalis*, *glabrata,* and *krusei* are implicated in gastric candidiasis, particularly in immunocompromised patients. Various risk factors were reported throughout these cases, including gastric acid suppression agents, immunosuppressive therapy, malignancy, previous surgery, end-stage liver disease, and diabetes mellitus. The most common complications included gastric ulceration, gastritis, and GI bleeding, though four cases reported severe complications of invasive gastric candidiasis such as perforation with one related fatality. Collectively, these studies are summarized in Table 2. Overall, our case adds to the available literature by highlighting the complex interplay between invasive gastric candidiasis, bacterial coinfections, recent antibiotic use, and related pathophysiology. To our knowledge, this is the first case that describes concurrent invasive gastric candidiasis, *Clostridioides difficile *colitis, and ESBL bacteremia.

**Table 2:** Summary of Reported Cases of Invasive Gastric Candidiasis. The table details patient demographics, comorbidities, risk factors, related complications, relevant coinfections, and *Candida spp.* involved. M, male; F, female.

Age/Sex	Risk Factors	Complication	Comorbidities	Coinfection	Candida spp.	Reference
58/M	Proton-Pump Inhibitors	Perforation	Hypertension	None	Candida tropicalis	[6]
50/M	Antacid Use	Perforation, Death	Smoking History	None	Candida albicans	[8]
74/M	Long-Term Corticosteroids, Cyclosporine	Perforation	Paroxysmal Nocturnal Hemoglobinuria	Pneumocystis jirovecii	Candida glabrata	[9]
70/M	None Identified	Perforation	Alcohol Use, Smoking History	None	Not Specified	[10]
54/F	End-Stage Liver Disease	Ulcers, Gastrointestinal Bleeding	Decompensated Cirrhosis	None	Candida albicans	[11]
59/M	Potassium Competitive Acid Blocker, Type 2 Diabetes Mellitus	Ulcers, Gastrointestinal Bleeding	History of *Helicobacter pylori*	None	Not Specified	[12]
54/F	Previous Cancer History with Abdominal Surgery	Gastric Dilatation, Necrotic Areas of Gastric Wall	Hypertension, Asthma, Rectal Cancer Surgery	None	Candida albicans	[13]
32/F	End-Stage Liver Disease, Immunosuppressive Therapy, Antibiotic Use	Gastric Wall Invasion with Mucosal Damage and Necrosis	Budd-Chiari Syndrome, Ascites, Esophageal Varices	Aspergillus fumigatus	Candida krusei	[14]
25/F	None Identified	Large Ulcer, Gastrointestinal Bleeding, Candidemia	Two Months Postpartum	None	Candida albicans	[15]
69/M	Subtotal Gastrectomy due to Gastric Cancer	Diffuse Ulcers, Stenosis at the Anastomotic Site	Type 2 Diabetes Mellitus	None	Not Specified	[16]
55/M	None Identified	Large Ulcer, Gastrointestinal Bleeding	Dyspepsia	Helicobacter pylori	*Candida albicans* and *Candida kefyr*	[17]
82/M	Elderly	Ulcers, Atrophic Gastritis	None	Helicobacter pylori	Not Specified	[18]
64/F	Cyclosporin A treatment	Ulcerated, Vegetating Gastric Lesion	Type 2 Diabetes Mellitus, Psoriasis	None	Candida albicans	[19]
73/M	Pancreatectomy	Gastrojejunostomy perforation	Pancreatic Adenocarcinoma	None	Not Specified	[20]
65/M	Pancreaticoduodenectomy	Gastrojejunostomy stenosis	Intra-ductal Papillary Mucinous Neoplasm	none	Not Specified	[20]

Pathophysiology

There are multiple explanations for the pathophysiological mechanisms of *Candida* overgrowth and resulting opportunistic pathogenicity leading to invasive gastric candidiasis. These mechanisms include mucosal barrier disruption, alterations of microbiota, disruptions in host immune responses, immune evasion, biofilm formation, and fungal morphogenic changes [21,22].

Intact GI epithelium is generally effective in preventing translocation or invasion of *Candida* into mucosal layers [23]. In the stomach, *Candida* has multiple adaptive advantages to allow for survivability, including proton pumps and stress-response pathways that allow for maintenance of an adequate intracellular pH [24]. Similarly, in normal gastric pH of ~1-2, *Candida* typically exists in its yeast form, while states of higher pH can facilitate hyphal transition [25]. Study findings have demonstrated that *Candida spp.,* including *C. albicans, C. parapsilosis, *and* C. tropicalis*, exhibit yeast-hyphal transition in simulated gastric fluid with elevated pH [26]. Quorum sensing, a phenomenon of intra-microbial communication, is also shown to control filamentation and pathogenic conversion between the dimorphic phases of *Candida* [27]. Hyphal transition is imperative for mucosal pathogenicity as hyphal proteins including hyphal wall protein 1, agglutinin-like sequence 3 in conjunction with secretion of aspartyl proteinases promote epithelial attachment and invasion [28]. Following invasion and transcellular translocation, fungal-mediated necrotic epithelial damage may be mediated by peptide toxins derived from hyphal protein Ece1 called candidalysin [29]. In our case, pathology did show hyphal morphology of the *Candida* indicating yeast to filamentous hyphal morphological switching occurred, likely playing a role in the observed ulcerations and necroinflammation.

Furthermore, the interplay between gastric microbiota and *Candida spp.* is dynamic, with important influences on pathogenicity, overgrowth, and immune signaling [30]. Elderly age and diabetes confer impairments in immune surveillance, facilitating weaker mucosal and innate immune responses, impairing fungal containment [31,32]. Microbial disruption via broad-spectrum antibiotic initiation or illness is a significant driver in overgrowth and pathogenic conversion of these fungi. In turn, reduction of bacterial diversity depletes bacteria taxa known to suppress *Candida spp.* through nutritional and resource competition and production of inhibitory metabolites such as short-chain fatty acids (SCFAs) [33,34]. For example, study findings have elucidated such mechanisms showing that microbiota-derived SCFAs can regulate mucosal immune homeostasis during *Candida* colonization by promoting IL-17A T-cell responses [35]. Furthermore, germ-free studies in mice have shown that gastric *Candida* promote inflammatory changes within one week of antibiotic-mediated depletion, highlighting the protective role of native microbiota [32]. Generally, microbial colonization in the stomach is comparatively lower than in other regions of the GI tract, with* Lactobacillus spp. *emerging as important players in preventing overgrowth of *Candida* throughout [36]. *Candida spp.* in gastric samples is shown to hinder the regrowth of beneficial bacterial species including Lactobacillus in the gastric mucosa and promote the growth of inflammatory genera such as *Enterococcus* [35]. Therefore, antibiotic depletion may promote longer-lasting effects contributing to pathogenic conversion of *Candida spp.* in gastric tissue. While there is no way to confirm these mechanisms specifically in our case, this data suggests that recent antibiotic use may have played a role in the development of invasive gastric candidiasis leading to multiple gastric ulcers seen on upper endoscopy.

*Candida spp.* also have adaptive advantages in the setting of acute illness and stress including metabolic flexibility through morphogenesis and potent nutrient acquisition mechanisms [37,38]. Furthermore, stress responses of *Candida* include ability to mitigate oxidative and nitrosative stress through utilization of transcription factors that can detoxify reactive oxygen and reactive nitrogen species [37]. This includes the high osmolarity glycerol response 1 (HOG1) and mitogen-activated protein kinase response pathway, which regulates glycerol synthesis to balance osmotic stress, promote formation of antioxidant enzymes, and induce heat-shock protein expression to preserve fungal viability [39]. Similarly, Candida AP-1 transcription factors (CAP1) also upregulate transcription of oxidative stress enzymes to promote survivability [40]. In sepsis, immune suppression and dysregulation with concomitant gut endothelial barrier disruption also allow for *Candida* translocation and invasion into mucosal layers [41]. Collectively, concurrent sepsis and ongoing acute illness likely amplified *Candida* invasion, permeating fungal invasion into the gastric mucosa and resulting in formation of the diffuse gastric ulcers seen in our case.

Lastly, the propensity of *Candida spp.* to form robust biofilms on gastric mucosa, composed of densely packed dimorphic fungi in an extracellular matrix, is a significant factor in the pathogenesis of invasive candidiasis [42]. Formation of these biofilms confers several advantages including protection against host immune defenses, low gastric pH, and antifungal therapy [43]. Predisposing factors to biofilm formation include broad-spectrum antibiotic use, immunosuppression and critical illness, and presence of medical devices [43]. No specific biofilm formation was identified on histopathological slides in our case; however, it is an important phenomenon that is worth mentioning and does not rule out the presence of biofilms in this patient.

Interactions between *Candida spp. *and *Clostridioides difficile*

Candidemia and *Clostridioides difficile* coinfection have been intricately linked, with rates of up to 10% of cases with candidemia demonstrating coexisting *Clostridium difficile *infection (CDI) within 90 days of diagnosis [44]. Around two out of three diagnoses of coinfection were made within one day apart with key risk factors including antibiotic use within 14 days (adjusted odds ratio 1.84) and hospitalization within 90 days (adjusted odds ratio 1.61) [44]. Furthermore, in a study with 29 CDI patients compared to *Clostridioides difficile* colonized controls, those with CDI were demonstrated to have statistically significantly higher abundances of *Candida spp. *[45]. Similarly, a study assessing risk factors for candidemia following CDI treatment found that severe CDI and CDI treatment with vancomycin and metronidazole increased the risk of developing candidemia [46]. Previous studies have also shown that 10-day treatment with metronidazole increased the GI and oropharyngeal colonization of *Candida spp.* [47]. This study's findings were supported by increased concentrations of *Candida spp.* in stool samples. In our case, metronidazole was given on admission one month prior to the presence of invasive gastric candidiasis for presumed GI infection, though the extent of contribution of this antibiotic is unclear. 

Recent studies have elucidated pathophysiological mechanisms linking GI candidiasis and CDI [48], potentially explaining the coinfection of these opportunistic bacterial species that contributed to mortality in our case. GI colonization of *Candida albicans *is shown to exacerbate CDI as *Candida* was shown to alter gut microbial composition and immune responses, augmenting *Clostridioides difficile *pathogenicity [49]. Depletion of *Candida* in this study mitigated these effects. Furthermore, murine studies have elucidated these interactions between CDI and candidiasis, through observations of worsening *Clostridioides difficile* disease severity with introduction of oral *Candida albicans* prior to infection [50]. These findings were shown through increased mortality, heightened intestinal permeability, and elevated concentrations of pro-inflammatory cytokines in these murine models. Notably, *Clostridioides difficile* bacterial load remained similar compared to controls that were not administered *Candida albicans*, indicating that *Candida* also modulates host immune factors rather than bacterial proliferation of *Clostridioides difficile*. Importantly, the introduction of probiotics consisting of *Bifidobacterium spp.* mitigated the severity of this coinfection in this study, suppressing both fecal *Candida* concentrations and pro-inflammatory cytokine response.

Furthermore, study findings have demonstrated that in the presence of *Candida albicans*, *Clostridioides difficile* can survive and grow under aerobic conditions, typically toxic to a strictly anaerobic bacterial species such as *Clostridioides* [51]. Interestingly, *Candida*-mediated oxygen scavenging may create localized hypoxic and relatively anaerobic microenvironments that facilitate *Clostridioides* pathogenicity and infection [51]. Taken together, these studies and mechanistic insights highlight compelling connections between *Candida spp. *and CDI, adding an interesting dynamic to our case.

Diagnosis

**Figure 5:**
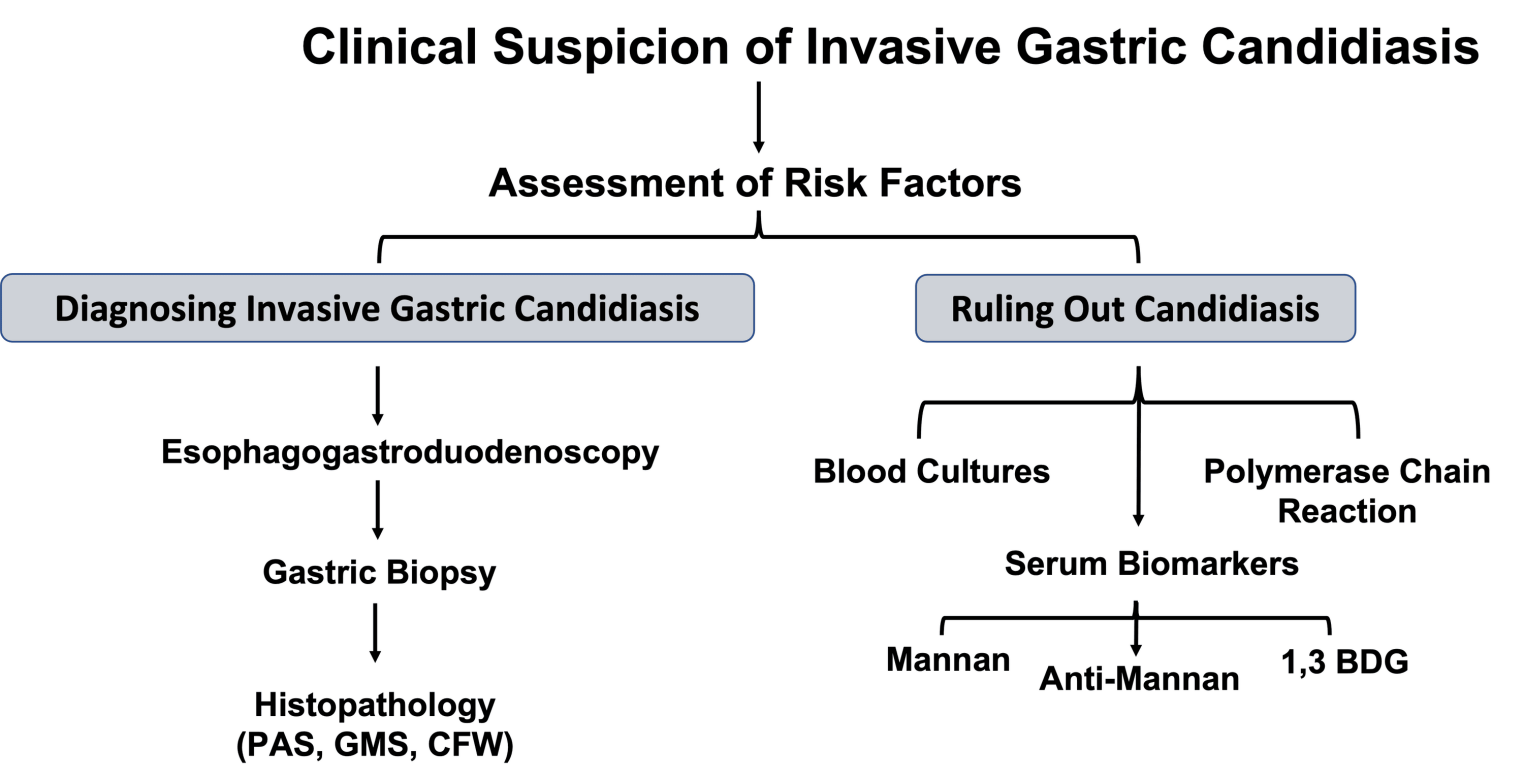
Diagnostic Pathway for Invasive Gastric Candidiasis and Ruling Out Candidiasis. PAS, Periodic Acid-Schiff; GMS, Grocott’s Methenamine Silver; CFW, Calcofluor White; 1,3 BDG, 1,3  β-D-glucan.

The diagnosis of invasive candidiasis should include assessment of risk factors, clinical pictures, and a combination of diagnostic testing (Figure 5).

However, diagnosis can be challenging due to nonspecific symptoms, presence of *Candida* in normal GI flora, sample contamination, and limitations due to low sensitivity [52]. For example, blood culture, typically the gold standard in diagnosing candidemia, has a sensitivity of between 50 and 70%, missing a considerable amount of bloodstream *Candida* infections [53]. Even then, blood cultures may take two to three days to grow, with a negative result not completely ruling out candidemia. In our case, blood cultures were never positive for *Candida* infection, delaying initial diagnosis.

Serum biomarkers are also used to identify *Candida* infection, particularly mannan, anti-Mannan, and 1,3 β-D-glucan (BDG) serve as useful indicators of disease, or lack thereof [54]. Mannans comprise the main cell wall component of *Candida spp.* [55], while BDG is present in the cell wall of most fungi [56], therefore less slightly specific in diagnosing *Candida*, but still useful in identifying fungal involvement. Anti-Mannan testing notably has 59% sensitivity with 93% specificity; however when combined with mannan testing, sensitivity increases to 83%, at the expense of specificity, which is slightly lower to 86% [57]. Sensitivities also differ based on *Candida spp.,* with *Candida albicans* being the most easily detectable when it comes to sensitivity [57]. For BDG assays, sensitivity in diagnosing candidemia was around 87.5%, while specificity was 85.5% [58]. Taken together, Mannan studies serve as more specific tests, while BDG assays are more sensitive [59]. In practice, these assays are often used in conjunction, and these biomarkers collectively are shown to have a high negative predictive value. Therefore, pan-negativity of these studies has strong utility in excluding *Candida* or other invasive fungal infections [60].

Polymerase chain reaction (PCR) of blood samples has the highest sensitivity and specificity in diagnosing candidemia [61]. Particularly, the Fungiplex Candida PCR, a multiplex PCR assay with the ability to identify multiple *Candida spp.*, showed excellent diagnostic yield, achieving 100% sensitivity and 94% specificity in serum samples from 58 ICU patients [62]. However, a limitation of PCR testing is that it does not differentiate between colonization from invasive infection, especially if samples are obtained from non-sterile sites such as the oropharynx or GI tract, where *Candida spp.* reside [63]. This can contribute to false positives and overtreatment of non-invasive *Candida* when taken from naturally colonized sources [63]. Nevertheless, PCR assays offer higher diagnostic accuracy and faster result times in diagnosing candidemia and deep-seated candidiasis when compared to serum biomarkers and blood cultures alone. This is demonstrated in a study that compared the sensitivities of diagnosing intra-abdominal, deep-seated candidiasis using PCR, BDG, and blood cultures, found to be 88%, 62% and 17%, respectively [64]. A recent study has suggested using a combination of more recently developed Wako β-D-glucan testing and CandID OLM RT-PCR testing, which when used in conjunction, achieve a 91% sensitivity and 100% specificity in 35 candidemia patients [65]. As such, a combination of serum biomarkers and PCR testing can significantly increase diagnostic acumen and should be considered.

Though the previously mentioned methods are useful in diagnosis, the mainstay of confirming diagnosis of invasive gastric candidiasis is histopathological [66], with gastric samples being obtained via esophagogastroduodenoscopy. Once tissue samples are obtained, Hematoxylin and eosin (H&E), PAS, Grocott’s methenamine silver (GMS) and Calcofluor White (CFW) stains assist in identifying *Candida* fungal elements [66]. H&E staining may assist in demonstrating fungal elements; however, PAS highlights the fungal wall in magenta and identifies morphology, while GMS staining can better delineate between yeast and hyphal forms, which serve a critical distinction in invasive candidiasis [62]. In recent years, CFW staining has offered advantages in diagnosing *Candida spp. *with higher sensitivity and specificity due to the color contrast that can offer clearer distinction of fungal elements from other surrounding structures [67]. In addition to identifying *Candida* morphological elements, diagnosis of invasive candidiasis requires penetration of mucosal layers, often into deeper tissue or tissue reactions, including necrosis, ulceration, and neutrophilic infiltration. These findings were clearly seen within the tissue biopsies obtained from gastric ulcer samples in our case, confirming the diagnosis of invasive gastric candidiasis. Though histopathology is an important diagnostic tool, limitations exist, including sampling error, patchy distribution of *Candida spp.*, lack of speciation, morphologic overlap, and misidentification of hyphal organisms [68].

Management

Management of invasive candidiasis, including candidiasis and deep-seated infections, involves initiation of antifungal medication, adequate duration of therapy, and source control, if warranted [69]. Echinocandins, such as caspofungin and micafungin, are first-line agents due to their broad coverage and inhibition of (1-->3)-β-D-glucan synthase of fungal cell membranes. For *Candida spp.*, echinocandins are fungicidal and cover a broad range of other fungal species that may also be involved. Generally, micafungin dosing starts at 100 mg intravenously daily and can be increased to 150mg IV daily in individuals with poor response. Caspofungin, on the other hand, requires a 70 mg loading dose, followed by 50 mg dosing daily. In non-neutropenic patients, fluconazole is an acceptable alternative as initial therapy, with options of intravenous or oral dosing, with loading of 800mg (~12 mg/kg), followed by 400 mg dosing daily (~6 mg/kg) [70]. In our case, fluconazole was initiated as the patient did not have risk factors for fluconazole resistance including previous azole treatment. It is also important to note that our patient did not necessarily have candidemia confirmed through blood cultures; however, patients with confirmed candidemia should have blood cultures repeated every other day until *Candida spp.* is cleared from the bloodstream [70]. Per the Infectious Disease Society of America (IDSA) guidelines, antifungal therapy should be continued for two weeks after documented clearance of candidemia and/or resolution of symptoms that are associated with candidemia [70]. In deep-seated invasive candidiasis, antifungal therapy duration is variable and can range from several weeks to months [69].

For cases of esophageal candidiasis, responses to micafungin are found to be dose-dependent with higher doses exerting a stronger antifungal effect [71]. 100-150 mg micafungin was also found to have a similar efficacy in clearing infection to 200 mg dosing of fluconazole in these patients with esophageal candidiasis [71]. It has also been suggested that for gastric candidiasis presenting as ulceration, PPI therapy without antifungal medication may be sufficient [72]. In a case series of 16 patients, benign localized ulcers not related to malignancy healed well with PPI alone [72]. However, the extent of ulceration seen in our case appeared to be far more extensive than the images seen in that case series, and antifungal therapy was warranted. In rare or severe cases of invasive gastric candidiasis leading to perforation, persistent bleeding due to ulceration, or malignant ulcers, surgery or endoscopic intervention may be required [72,73].

There are no specific guidelines on repeat endoscopy specifically for ulcers mediated by *Candida spp.* However, the American Society of Gastrointestinal Endoscopy (ASGE) recommends repeating surveillance endoscopies for gastric ulcers due to a higher risk of malignancy [74]. However, risk stratification should be performed, and decision to repeat endoscopy should be individualized. In our patient with significant ulcerations, recommendations were to repeat endoscopy to confirm resolution; however, the patient passed away before this could be performed.

Outcomes

Despite advancements in diagnosis and initiation of adequate therapy, mortality rates remain high [75]. Invasive candidiasis, including candidemia and deep-seated candidiasis, ranges from 40 to 55% [76,77]. High mortality rates can be attributed to multiple factors discussed throughout this text, including diagnostic limitations, antimicrobial resistance, biofilm formation, inadequate treatment, and the presence of invasive candidiasis in high-risk individuals with severe comorbidities.

As such, to improve patient outcomes, early initiation of empiric antifungal therapy within 24 hours of suspected invasive candidiasis, source control when relevant, and treatment of comorbid factors is imperative. Similarly, atypical appearing ulcers in the upper GI tract should be biopsied for evaluation of invasive candidiasis in patients with risk factors, with consideration of initiating antifungal therapy if patients are not improving while awaiting biopsy results. Atypical gastric ulcers include those on the greater curvature of the antrum, gastric cardia, or gastric fundus [78]. Concerning features include irregular, heaped-up margins, necrotic appearance, and raised edges. Multiple, large ulcers and ulcers refractory to treatment are also concerning for worrying pathology.

However, initiation of broad-spectrum antifungals should be used in those with high clinical suspicion, as widespread use may contribute to antifungal resistance, worsening future outcomes [79]. Important antifungal stewardship strategies geared towards reducing invasive *Candida* infections include implementation of evidence-based guidelines, optimization of diagnostic tools, as well as prompt de-escalation and stepdown strategies [80]. Local antifungal resistance patterns should also be reviewed, and infectious disease specialist consultation to help guide antimicrobial choice is beneficial, particularly in the setting of intravenous antifungal use.

## Conclusions

Diagnosing gastrointestinal *Candida* infections remains challenging, as fungal pathogens are often overlooked in abdominal pathology, and blood cultures have limited sensitivity for detecting intra-abdominal *Candida spp.* In this case, timely upper endoscopy and biopsy were essential for confirming the diagnosis and guiding antifungal therapy. Histopathological findings of fungal pseudohyphae adjacent to necrotic tissue strongly support the presence of a pathologic infection rather than mere colonization. This distinction is crucial, as pathologic gastric candidiasis is associated with significant morbidity and mortality, requiring prompt recognition and appropriate treatment. Importantly, antimicrobial stewardship is an important factor in pathologic conversion of *Candida spp.* particularly due to the mechanistic interplay with *Clostridioides difficile *coinfection. This case highlights the importance of maintaining a high index of suspicion for fungal infections in patients with large atypical appearing gastric ulcers, even in the absence of overt immunosuppression.
